# Supplementary material for: Engineering Therapeutic Strategies in Cancer Immunotherapy via Exogenous Delivery of Toll-like Receptor Agonists
Source: Pharmaceutics. 2021 Aug 31;13(9):1374. doi: 10.3390/pharmaceutics13091374 (PMC8466827; doi:10.3390/pharmaceutics13091374)
Supplement: Supplementary file 1 [file pharmaceutics-13-01374-s001.zip › pharmaceutics-1297575-supplementary.pdf]

pharmaceutics

MDPI

Supplementary Material: Engineering Therapeutic Strategies in Cancer Immunotherapy via Exogenous Delivery of Toll-Like Receptor Agonists

Sehwan Jeong, Yunyoung Choi and Kyobum Kim

Table S1. Current clinical trials of TLR agonists with or without co-factors on cancer therapy.

TLR	TLR agonist	Phase	Co-factors	Conditions	Status	Ref.
3	PolyICLC	I	Romidepsin, Focal Lesional Radiation	Cutaneous T-cell Lymphoma	Terminated	NCT02061449
		I, II	NY-ESO-1 protein, Montanide	Melanoma	Completed	NCT01079741
		I	OC-L, Montanide	Primary Ovarian Cancer, Fallopian Tube Cancer, Primary Peritoneal Cancer	Terminated	NCT02452775
		I, II	Durvalumab, Tremelimumab	Head and Neck Squamous Cell Carcinoma, Breast Cancer and 9 more	Active, not recruiting	NCT02643303
		I, II	rhuFlt3L/CDX-301	Low-Grade B-cell Lymphoma	Recruiting	NCT01976585
		I, II	6MHP, NeoAg-mBRAF, CDX-1140	Melanoma	Recruiting	NCT04364230
	Rintatolimod	I, II	Pembrolizumab, Cisplatin	Ovarian Cancer Recurrent	Recruiting	NCT03734692
4	G100	I, II	Pembrolizumab, Rituximab	Follicular Low Grade Non-Hodgkin's Lymphoma	Terminated	NCT02501473
	GLA-SE	I	.	Stage III/IV Adult Soft Tissue Sarcoma,	Completed	NCT02180698
		I	MART-1 antigen	Stage IIA/IIB/IIB/IIIA/IIIB/IIIC/IV Skin Melanoma	Completed	NCT02320305
	GSK179509	I	.	Cancer, Neoplasms	Completed	NCT02798978
5	Entolimod	I	.	Unspecified Adult Solid Tumor, Protocol Specific	Completed	NCT01527136
7	BNT411	I, II	Atezolizumab (anti-PD-1), Carboplatin, Etoposide	Solid Tumor, Extensive-stage Small Cell Lung Cancer	Recruiting	NCT04101357
	DSP-0509	I, II	Pembrolizumab	Neoplasms	Recruiting	NCT03416335
	Imiquimod	II	.	Breast Cancer, Breast Neoplasms	Completed	NCT00899574
		I	Indocyanine green solution	Melanoma, Metastatic Cancer	Completed	NCT00453050
		I	Cyclophosphamide	Breast Cancer, Metastatic Breast Cancer, Recurrent Breast Cancer	Completed	NCT01421017
		I	Standard of Care PD-1 Therapy	Melanoma, Breast Cancer, Merkel Cell Carcinoma and 10 more	Recruiting	NCT04116320
	RO7119929	I	Tocilizumab	Carcinoma, Hepatocellular, Biliary Tract cancer, Secondary Liver Cancer, Liver Metastases	Recruiting	NCT04338685
	SHR2150	I, II	anti-PD-1 antibody, anti-CD47	Solid Tumor	Recruiting	NCT04588324
8	VTX-2337	I, II	.	Low Grade B Cell Lymphoma	Terminated	NCT01289210
		I	PLDH, Paclitaxel	Malignant Ovarian Mixed Epithelial Tumor, Ovarian Brenner Tumor and 8 more	Completed	NCT01294293
		I	Cetuximab	Metastatic Squamous Neck Cancer With Occult Primary Squamous Cell Carcinoma, Recurrent Metastatic Squamous Neck Cancer With Occult Primary and 39 more	Completed	NCT01334177
		II	PLD	Epithelial Ovarian Cancer, Fallopian Tube Cancer, Primary Peritoneal Cancer	Completed	NCT01666444
		II	Carboplatin, Cisplatin, 5-fluorouracil	Carcinoma, Squamous Cell of Head and Neck	Completed	NCT01836029

		I	Cyclophosphamide, Pegfilgrastim	Colorectal Adenocarcinoma, Metastatic Pancreatic Adenocarcinoma and 16 more	Terminated	NCT02650635
		I	Nivolumab	Carcinoma, Squamous Cell	Recruiting	NCT03906526
9	CMP-001	II	Nivolumab	Melanoma, Lymph Node Cancer	Active, not recruiting	NCT03618641
		II	Nivolumab	Melanoma	Recruiting	NCT04401995
	CPG 7909	I, II	.	Non-Hodgkin Lymphoma, Mycosis Fungoides	Completed	NCT00185965
	EMD 1201081	I	Cisplatin, Cetuximab	Squamous Cell Carcinoma of the Head and Neck	Terminated	NCT01360827
	GNKG168	I	.	Relapsed Acute Lymphoblastic Leukemia, Relapsed Acute Myelogenous Leukemia	Terminated	NCT01743807
	MGN1703	I	Ipilimumab	Advanced Cancers, Melanoma	Active, not recruiting	NCT02668770
	SD-101	I, II	Ipilimumab	Extranodal Marginal Zone Bcell Lymphoma of Mucosa-associated Lymphoid Tissue, Nodal Marginal Zone B-cell Lymphoma, and 5 more	Completed	NCT02254772
		I	BMS 986178 (Anti-OX40 Antibody)	Advanced Malignant Solid Neoplasm, Extracranial Solid Neoplasm, Metastatic Malignant Solid Neoplasm	Active, not recruiting	NCT03831295
		I	Anti-OX40 Antibody BMS 986178	B-Cell Non-Hodgkin Lymphoma, Grade 1/2/3a Follicular Lymphoma and 4 more	Recruiting	NCT03410901
		I	Nivolumab	Metastatic Pancreatic Adenocarcinoma, Refractory Pancreatic Adenocarcinoma, Stage IV Pancreatic Cancer AJCC v8	Recruiting	NCT04050085
		I	Nivolumab, Ipilimumab	Metastatic Uveal Melanoma in the Liver	Recruiting	NCT04935229
		I, II	Ibrutinib	Grade 1/2/3a Follicular Lymphoma, Recurrent Follicular Lymphoma and 3 more	Recruiting	NCT02927964
	Tilsotolimod	I	Ipilimumab, Nivolumab	Advanced Cancer	Active, not recruiting	NCT04270864
	VLP-encapsulated CMP-001	II	Pembrolizumab	Clinical Stage III Cutaneous Melanoma AJCC v8, Melanoma of Unkown Primary and 4 more	Recruiting	NCT04708418
		I, II	INCAGN01949	Locally Advanced Malignant Solid Neoplasm, Metastatic Pancreatic Adenocarcinoma and 2 more	Not yet recruiting	NCT04387071
3, 7/8	PolyICLC, R848	I, II	Peptide Vaccine (LPV7) + Tetanus peptide, IFA	Melanoma, metastatic melanoma, mucosal melanoma	Active, not recruiting	NCT02126579
		II	Tumorlysate pulsed DC vaccination	Glioma, Anaplastic Astrocytoma, Anaplastic Astro-oligodendroglioma, Glioblastoma	Active, not recruiting	NCT01204684
7/8	BDB018	I	Pembrolizumab	Solid Tumor	Recruiting	NCT04840394
	BDC-1001	I, II	Pembrolizumab	HER2 Positive Solid Tumors	Recruiting	NCT04278144
	MEDI9197	I	Durvalumab	Solid Tumors, CTCL, Cancer	Terminated	NCT02556463
	R848	I	.	Tumors	Completed	NCT00821652
		II	Gp100, MAGE-3	Melanoma	Completed	NCT00960752
	TransCon	I, II	Pembrolizumab	Advanced Solid tumor, Locally Advanced Solid Tumor, Metastatic Solid Tumor	Recruiting	NCT04799054

MART-1, melanoma antigen recognized by T-cells; R848, resiquimod; PLD, pegylated liposomal doxorubicin; Pegylated Liposomal Doxorubicin Hydrochloride (PLDH); VLP, virus-like particles; AJCC, American Joint Committee on Cancer.
